# 1-(4-Chloro­benzyl­idene)-4-ethyl­thio­semicarbazide

**DOI:** 10.1107/S1600536810047446

**Published:** 2010-11-20

**Authors:** Yu-Feng Li

**Affiliations:** a Microscale Science Institute, Department of Chemistry and Chemical Engineering, Weifang University, Weifang 261061, People’s Republic of China

## Abstract

In the title compound, C_10_H_12_ClN_3_S, the dihedral angle between the benzene ring and the thio­urea unit is 2.35 (19)°. In the crystal, inversion dimers linked by pairs of N—H⋯S hydrogen bonds generate *R*_2_^2^(8) loops.

## Related literature

For related structures, see: Li & Jian (2010 ▶); Li & Meng (2010 ▶).
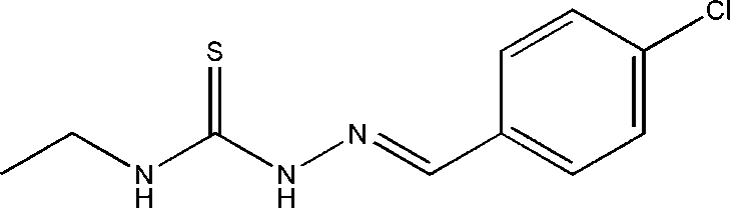


## Experimental

### 

#### Crystal data

C_10_H_12_ClN_3_S
*M* *_r_* = 241.75
Monoclinic, 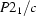

*a* = 4.6769 (10) Å
*b* = 26.727 (6) Å
*c* = 9.791 (3) Å
β = 102.59 (3)°
*V* = 1194.4 (5) Å^3^
*Z* = 4
Mo *K*α radiation
μ = 0.47 mm^−1^
*T* = 293 K
0.22 × 0.20 × 0.18 mm

#### Data collection

Bruker SMART CCD diffractometer
11437 measured reflections
2723 independent reflections
1388 reflections with *I* > 2σ(*I*)
*R*_int_ = 0.066

#### Refinement

*R*[*F* ^2^ > 2σ(*F* ^2^)] = 0.068
*wR*(*F* ^2^) = 0.272
*S* = 1.09
2723 reflections
136 parameters
H-atom parameters constrained
Δρ_max_ = 0.33 e Å^−3^
Δρ_min_ = −0.34 e Å^−3^

### 

Data collection: *SMART* (Bruker, 1997 ▶); cell refinement: *SAINT* (Bruker, 1997 ▶); data reduction: *SAINT*; program(s) used to solve structure: *SHELXS97* (Sheldrick, 2008 ▶); program(s) used to refine structure: *SHELXL97* (Sheldrick, 2008 ▶); molecular graphics: *SHELXTL* (Sheldrick, 2008 ▶); software used to prepare material for publication: *SHELXTL*.

## Supplementary Material

Crystal structure: contains datablocks global, I. DOI: 10.1107/S1600536810047446/hb5737sup1.cif

Structure factors: contains datablocks I. DOI: 10.1107/S1600536810047446/hb5737Isup2.hkl

Additional supplementary materials:  crystallographic information; 3D view; checkCIF report

## Figures and Tables

**Table 1:** Hydrogen-bond geometry (Å, °)

*D*—H⋯*A*	*D*—H	H⋯*A*	*D*⋯*A*	*D*—H⋯*A*
N2—H2*A*⋯S1^i^	0.86	2.59	3.383 (4)	154

Symmetry code: (i) 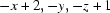.

**Fig. 1.**
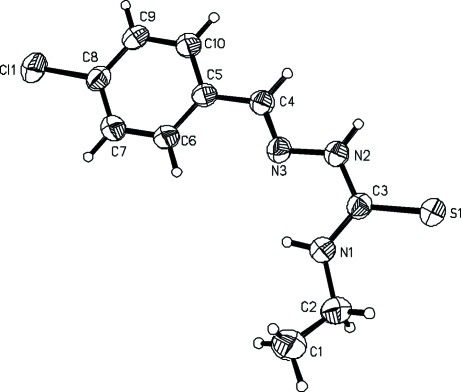
The structure of the title compound showing 30% probability displacement ellipsoids.

## supplementary crystallographic information

### Experimental

A mixture of 4-ethylthiosemicarbazide (0.1 mol) and 4-chlorobenzaldehyde (0.1 mol) was stirred in refluxing ethanol (20 mL) for 2 h to afford the title compound (0.089 mol, yield 89%).  Colourless bars were obtained by recrystallization from ethanol at room temperature.

### Refinement

H atoms were fixed geometrically and allowed to ride on their attached atoms, with C—H distances=0.97 Å, and with *U*_iso_=1.2–1.5*U*_eq_.

### Crystal data

**Table:**

C_10_H_12_ClN_3_S	*F*(000) = 504
*M**_r_* = 241.75	*D*_x_ = 1.344 Mg m^−^^3^
Monoclinic, *P*2_1_/*c*	Mo *K*α radiation, λ = 0.71073 Å
Hall symbol: -P 2ybc	Cell parameters from 2723 reflections
*a* = 4.6769 (10) Å	θ = 3.1–27.5°
*b* = 26.727 (6) Å	µ = 0.47 mm^−^^1^
*c* = 9.791 (3) Å	*T* = 293 K
β = 102.59 (3)°	Bar, colorless
*V* = 1194.4 (5) Å^3^	0.22 × 0.20 × 0.18 mm
*Z* = 4	

### Data collection

**Table:**

Bruker SMART CCD diffractometer	1388 reflections with *I* > 2σ(*I*)
Radiation source: fine-focus sealed tube	*R*_int_ = 0.066
graphite	θ_max_ = 27.5°, θ_min_ = 3.1°
phi and ω scans	*h* = −6→5
11437 measured reflections	*k* = −34→34
2723 independent reflections	*l* = −12→12

### Refinement

**Table:**

Refinement on *F*^2^	Primary atom site location: structure-invariant direct methods
Least-squares matrix: full	Secondary atom site location: difference Fourier map
*R*[*F*^2^ > 2σ(*F*^2^)] = 0.068	Hydrogen site location: inferred from neighbouring sites
*wR*(*F*^2^) = 0.272	H-atom parameters constrained
*S* = 1.09	*w* = 1/[σ^2^(*F*_o_^2^) + (0.1156*P*)^2^ + 1.1747*P*] where *P* = (*F*_o_^2^ + 2*F*_c_^2^)/3
2723 reflections	(Δ/σ)_max_ < 0.001
136 parameters	Δρ_max_ = 0.33 e Å^−^^3^
0 restraints	Δρ_min_ = −0.33 e Å^−^^3^

### Special details

**Table:**

Geometry. All e.s.d.'s (except the e.s.d. in the dihedral angle between two l.s. planes) are estimated using the full covariance matrix. The cell e.s.d.'s are taken into account individually in the estimation of e.s.d.'s in distances, angles and torsion angles; correlations between e.s.d.'s in cell parameters are only used when they are defined by crystal symmetry. An approximate (isotropic) treatment of cell e.s.d.'s is used for estimating e.s.d.'s involving l.s. planes.
Refinement. Refinement of *F*^2^ against ALL reflections. The weighted *R*-factor *wR* and goodness of fit *S* are based on *F*^2^, conventional *R*-factors *R* are based on *F*, with *F* set to zero for negative *F*^2^. The threshold expression of *F*^2^ > σ(*F*^2^) is used only for calculating *R*-factors(gt) *etc*. and is not relevant to the choice of reflections for refinement. *R*-factors based on *F*^2^ are statistically about twice as large as those based on *F*, and *R*- factors based on ALL data will be even larger.

### Fractional atomic coordinates and isotropic or equivalent isotropic displacement parameters (Å^2^)

**Table:**

	*x*	*y*	*z*	*U*_iso_*/*U*_eq_	
S1	1.2104 (3)	−0.01352 (5)	0.72223 (14)	0.0738 (5)	
Cl1	−0.3450 (4)	0.27875 (6)	0.46099 (18)	0.1022 (6)	
N2	0.7928 (8)	0.04912 (15)	0.6097 (4)	0.0637 (10)	
H2A	0.7695	0.0311	0.5354	0.076*	
N3	0.6207 (8)	0.09071 (14)	0.6128 (4)	0.0616 (10)	
C5	0.2251 (9)	0.14199 (17)	0.4970 (5)	0.0576 (11)	
C4	0.4166 (10)	0.09865 (18)	0.5041 (5)	0.0622 (11)	
H4A	0.3907	0.0765	0.4291	0.075*	
N1	1.0195 (10)	0.06662 (17)	0.8346 (4)	0.0789 (13)	
H1A	0.8985	0.0912	0.8260	0.095*	
C10	0.0157 (10)	0.15053 (19)	0.3771 (5)	0.0664 (12)	
H10A	−0.0066	0.1281	0.3028	0.080*	
C6	0.2528 (11)	0.1751 (2)	0.6072 (5)	0.0723 (13)	
H6A	0.3933	0.1695	0.6889	0.087*	
C9	−0.1627 (11)	0.1925 (2)	0.3665 (5)	0.0709 (13)	
H9A	−0.3045	0.1983	0.2853	0.085*	
C3	0.9998 (10)	0.03689 (17)	0.7250 (5)	0.0602 (11)	
C8	−0.1287 (11)	0.22552 (19)	0.4764 (5)	0.0679 (12)	
C7	0.0731 (14)	0.2164 (2)	0.5967 (6)	0.0836 (16)	
H7A	0.0898	0.2382	0.6721	0.100*	
C2	1.2242 (16)	0.0617 (2)	0.9669 (6)	0.096 (2)	
H2B	1.1446	0.0383	1.0247	0.116*	
H2C	1.4045	0.0475	0.9506	0.116*	
C1	1.290 (2)	0.1061 (3)	1.0404 (9)	0.130 (3)	
H1B	1.4287	0.0997	1.1264	0.196*	
H1C	1.1142	0.1199	1.0606	0.196*	
H1D	1.3723	0.1294	0.9851	0.196*	

### Atomic displacement parameters (Å^2^)

**Table:**

	*U*^11^	*U*^22^	*U*^33^	*U*^12^	*U*^13^	*U*^23^
S1	0.0781 (9)	0.0700 (8)	0.0700 (8)	0.0180 (6)	0.0089 (6)	0.0022 (6)
Cl1	0.1142 (13)	0.0852 (11)	0.1023 (12)	0.0396 (9)	0.0129 (9)	0.0139 (8)
N2	0.060 (2)	0.069 (2)	0.061 (2)	0.0125 (19)	0.0087 (17)	−0.0022 (18)
N3	0.060 (2)	0.062 (2)	0.063 (2)	0.0073 (19)	0.0145 (18)	0.0012 (17)
C5	0.054 (2)	0.062 (3)	0.055 (2)	−0.002 (2)	0.0104 (19)	0.0026 (19)
C4	0.057 (3)	0.069 (3)	0.059 (3)	0.008 (2)	0.010 (2)	−0.001 (2)
N1	0.087 (3)	0.079 (3)	0.063 (3)	0.025 (2)	0.000 (2)	−0.008 (2)
C10	0.063 (3)	0.070 (3)	0.062 (3)	0.002 (2)	0.006 (2)	−0.001 (2)
C6	0.072 (3)	0.077 (3)	0.060 (3)	0.014 (3)	−0.003 (2)	−0.004 (2)
C9	0.066 (3)	0.077 (3)	0.064 (3)	0.009 (3)	0.001 (2)	0.013 (2)
C3	0.057 (2)	0.063 (3)	0.062 (3)	0.006 (2)	0.013 (2)	0.005 (2)
C8	0.068 (3)	0.067 (3)	0.067 (3)	0.011 (2)	0.011 (2)	0.012 (2)
C7	0.098 (4)	0.079 (4)	0.068 (3)	0.025 (3)	0.005 (3)	−0.011 (3)
C2	0.111 (5)	0.085 (4)	0.077 (4)	0.012 (4)	−0.014 (3)	−0.005 (3)
C1	0.153 (7)	0.094 (5)	0.112 (6)	−0.002 (5)	−0.039 (5)	−0.022 (4)

### Geometric parameters (Å, °)

**Table:**

S1—C3	1.673 (5)	C10—H10A	0.9300
Cl1—C8	1.733 (5)	C6—C7	1.379 (7)
N2—C3	1.357 (6)	C6—H6A	0.9300
N2—N3	1.377 (5)	C9—C8	1.373 (7)
N2—H2A	0.8600	C9—H9A	0.9300
N3—C4	1.283 (6)	C8—C7	1.361 (7)
C5—C10	1.374 (6)	C7—H7A	0.9300
C5—C6	1.379 (7)	C2—C1	1.386 (9)
C5—C4	1.457 (6)	C2—H2B	0.9700
C4—H4A	0.9300	C2—H2C	0.9700
N1—C3	1.322 (6)	C1—H1B	0.9600
N1—C2	1.439 (7)	C1—H1C	0.9600
N1—H1A	0.8600	C1—H1D	0.9600
C10—C9	1.389 (7)		
			
C3—N2—N3	119.4 (4)	N1—C3—N2	116.2 (4)
C3—N2—H2A	120.3	N1—C3—S1	124.1 (4)
N3—N2—H2A	120.3	N2—C3—S1	119.7 (4)
C4—N3—N2	116.6 (4)	C7—C8—C9	120.3 (5)
C10—C5—C6	119.3 (4)	C7—C8—Cl1	120.2 (4)
C10—C5—C4	119.3 (4)	C9—C8—Cl1	119.5 (4)
C6—C5—C4	121.4 (4)	C8—C7—C6	120.2 (5)
N3—C4—C5	120.6 (4)	C8—C7—H7A	119.9
N3—C4—H4A	119.7	C6—C7—H7A	119.9
C5—C4—H4A	119.7	C1—C2—N1	114.7 (6)
C3—N1—C2	126.2 (5)	C1—C2—H2B	108.6
C3—N1—H1A	116.9	N1—C2—H2B	108.6
C2—N1—H1A	116.9	C1—C2—H2C	108.6
C5—C10—C9	120.2 (5)	N1—C2—H2C	108.6
C5—C10—H10A	119.9	H2B—C2—H2C	107.6
C9—C10—H10A	119.9	C2—C1—H1B	109.5
C5—C6—C7	120.3 (5)	C2—C1—H1C	109.5
C5—C6—H6A	119.9	H1B—C1—H1C	109.5
C7—C6—H6A	119.9	C2—C1—H1D	109.5
C8—C9—C10	119.7 (4)	H1B—C1—H1D	109.5
C8—C9—H9A	120.2	H1C—C1—H1D	109.5
C10—C9—H9A	120.2		

### Hydrogen-bond geometry (Å, °)

**Table:**

*D*—H···*A*	*D*—H	H···*A*	*D*···*A*	*D*—H···*A*
N2—H2A···S1^i^	0.86	2.59	3.383 (4)	154

Symmetry codes: (i) −*x*+2, −*y*, −*z*+1.
